# Altered connection properties of important network hubs may be neural risk factors for individuals with primary insomnia

**DOI:** 10.1038/s41598-018-23699-3

**Published:** 2018-04-12

**Authors:** Xuming Liu, Jiyong Zheng, Bi-Xia Liu, Xi-Jian Dai

**Affiliations:** 1 Department of Radiology, The Third Clinical Institute Affiliated to Wenzhou Medical University, Wenzhou, 325000 Zhejiang People’s Republic of China; 2 Department of Medical Imaging, The affiliated Huaian No. 1 People’s Hospital of Nanjing Medical University, Huai’an, 223300 Jiangsu People’s Republic of China; 3 Department of Respiration, the First Hospital Affiliated to Soochow University, Suzhou, 215006 People’s Republic of China; 4 Department of ICU, Jiangxi Provincial Cancer Hospital, Nanchang, 330029 Jiangxi People’s Republic of China; 5 Department of Medical Imaging, Jinling Hospital, Medical School of Nanjing University, Nanjing, 210002 Jiangsu People’s Republic of China; 6 Department of Radiology, The First Affiliated Hospital of Nanchang University, Nanchang, 330006 Jiangxi People’s Republic of China

## Abstract

Primary insomnia (PIs) is highly prevalent and can lead to adverse socioeconomic impacts, but the underlying mechanism of its complex brain network impairment remains largely unknown. Functional studies are too few and diverse in methodology, which makes it difficult to glean general conclusions. To answer this question, we first used graph theory-based network analyse, together with seed-based functional connectivity approach, to characterize the topology architecture of whole-brain functional networks associated with PIs. Forty-eight subjects with PIs and 48 age/sex/education-matched good sleepers were recruited. We found PIs is associated with altered connection properties of intra-networks within the executive control network, default mode network and salience network, and inter-network between auditory language comprehension center and executive control network. These complex networks were correlated with negative emotions and insomnia severity in the PIs group. Altered connection properties of these network hubs appeared to be neural risk factors for neuropsychological changes of PIs, and might be used as potential neuroimaging markers to distinguish the PIs from the good sleepers. These findings highlight the role of functional connectivity in the pathophysiology of PIs, and may underlie the neural mechanisms of etiology of PIs.

## Introduction

Each of us spend almost a third of our life asleep. Precise control of the sleep process is the basis of normal life process including blood, metabolism, immune, endocrine, and brain activity, and is key to plasticity formation, information processing, and function implementation^1–4^. Therefore, elucidation of the neurobiological effects of sleep and waking remain an important goal of basic and clinical neurosciences. Insomnia is a highly prevalent sleep complaint affecting 10–15% of the adult population^5^. Patients with primary insomnia (PIs) have the subjective experience of chronically disturbed sleep, sleep loss, non-refreshing sleep, and heightened arousal in bed. These conditions can adversely affect social, emotional, and cognitive behavior, psychomotor performance, and metabolism, and can even lead to multi-systemic and multi-organ dysfunction^6–14^. Despite the adverse socioeconomic impacts of primary insomnia, its neurobiological causes and consequences remain elusive. Considering the high prevalence of insomnia and its known neurotoxic effects, better understanding of the complex brain network impairments underlying the condition is essential; however, the mechanisms causing these neuropsychiatric alterations remain ambiguous. Therefore, elucidation of the neurobiological changes associated with primary insomnia has the potential to provide insights into cognitive and emotional changes, and bridge the gap between sleep loss and neurological or psychiatric disorders.

Numerous neurobiological studies have reported metabolic, morphological, and functional brain alterations associated with primary insomnia. Regarding morphological neuroimaging aspects, a number of studies have been exclusively dedicated to addressing the brain structural features associated with insomnia; however, it remains difficulties to determine a consistent explanation for the neuropathology of brain microstructure alterations in the condition, since the results of structural studies are either contradictory or require replication^15–23^. Moreover, functional neuroimaging studies are scarce and have used diverse methodologies, preventing the development of general conclusions^24,25^.

Seed-based functional connectivity studies have revealed abnormal connectivity patterns in PIs in brain regions related to emotion and cognition^26–29^; however, the seed-based functional connectivity analysis provides limited information about the relationships between a given seed point region and other brain regions in a whole brain network. Independent component analysis can not evaluate the strength of functional connectivity among brain regions. Recently, graph theory-based network analysis has been applied to explore brain connectivity within whole-brain networks. Specifically, voxel-wise degree centrality is a type of graph-theoretic measurement that assesses the topology of the architecture of the brain functional connectome at the voxel level, with each voxel treated as an independent node, and represents the number of direct connections for a given voxel in a voxel-wise connectome^30–32^. In contrast to the traditional seed-based functional connectivity, degree centrality analysis, based on voxel-based whole-brain correlation analysis, provides an opportunity for unbiased searches abnormalities within the entire connectivity matrix of the full-brain functional connectome without the need for a priori hypothesis, and does not require a priori definition of regions of interest (ROIs). The degree centrality analysis can measure the importance of individual nodes and may reflect the information flow characteristics of functional brain network “hub” properties (i.e., provide network information^33^) with relatively high test-retest reliability^34^. Therefore, the degree centrality analysis can make up for the lack of traditional functional connectivity analysis. Recently the degree centrality analysis has been successfully used to disclose the neurobiological mechanism underlying several diseases, including obsessive compulsive disorder^35^, Alzheimer’s disease^36^ and major depressive disorder^37^; however, primary insomnia has not previously been studied.

Primary insomnia is associated with changes in behavior, brain function, and brain structure; however, the nature of these changes is not well understood. The combination of the neuroimaging and the sleep and emotional assessment has the potential to elucidate the biological mechanisms underlying insomnia and lead to the development of improved treatment strategies. In this study, we hypothesized that individuals with primary insomnia may exhibit impaired connectivity patterns in emotional and cognitive-related regions. To provide a new insight into the neurobiological mechanisms underlying primary insomnia, we report the first use of a voxel-wise degree centrality approach to identify altered intrinsic functional connectivity hubs, based on voxel-based whole-brain correlation analysis, from the entire connectivity matrix of full-brain functional connectomes. Although voxel-wise degree centrality analysis can identify voxels with altered functional connectivity with other voxels, it cannot reflect the interactions between a given seed point region and other specific regions. Thus, brain regions exhibiting abnormal degree centrality were saved as ROIs and used for further resting-state functional connectivity analysis to obtain additional information about the connectivity patterns in primary insomnia. Next, we conducted multiple linear regression analysis to evaluate the relationships between behavioral factors and degree centrality values of significant alterations in intrinsic functional hubs, and between behavioral factors and strength of functional connectivity of paird functional hubs. The intraclass correlation coefficient (ICC) is a common index of test-retest reliability that ranges from 0 (no reliability) to 1 (perfect reliability). In this study, we also used the ICC to investigate the test-retest stability of degree centrality measurements, as described in our previous study^24^.

## Materials and Methods

### Subjects

This study was approved by the Medical Research Ethical Committee of Jinling Hospital of Medical School of Nanjing University (Nanjing, China) in accordance with the Declaration of Helsinki, and written informed consent was obtained from all subjects. A total of 48 PIs (32 female, 16 male; mean age, 46.48 ± 12.6 years; mean ± std) and 48 age-, sex-, and education-matched good sleepers (GSs; 25 female, 23 male; mean age, 45.69 ± 12.53 years; mean ± std) were recruited from the hospital and the community. Of those patients, 25 PIs (8 male, 17 female) were not first-time visitors and had previously taken hypnotic or psychoactive medication. The other 23 PIs (8 male, 15 female) were first-time visitors and had never taken medication before. To avoid the possible effect of medication, PIs were kept medication-free for at least two weeks prior to data collection and for the duration of this study, except that three PIs were medication-free for only 2–3 days. The mean duration of insomnia for PIs was (4.96 ± 5.25 years; mean ± std).

The PIs met the relevant diagnostic criteria of the International Classification of Sleep Disorders (third Edition), Pittsburgh Sleep Quality Index (PSQI) score> 5, and sleep diary for> 2 weeks duration. Furthermore, they had to report a total sleep time ≤ 6.5 h and (a) sleep onset latency> 45 min or (b) wake after sleep onset> 45 min or (c) total wake time during the sleep period (sleep latency + wake after sleep onset)> 60 min. To evaluate their sleep status, PIs were asked to wear a Fitbit Flex tracker (http://help.fitbit.com) for two consecutive nights, and GSs were asked to wear the tracker for one consecutive week^24^. These data were primarily used to verify sleep-wake diary information and not for independent assessment of inclusion and exclusion criteria.

All GSs met the following criteria: good sleeping habits, good sleep onset (<30 min) and/or maintenance (without easily wakened or morning awakening symptom) and regular dietary habits as measured by the Fitbit Flex tracker and sleep diary; no consumption of any stimulants, hypnotic or psychoactive medication, during or prior to the study for ≥3 months; PSQI score <5, and Hamilton Depression Rating Scale (HAMD) and Hamilton Anxiety Rating Scale (HAMA) <7. All subjects were right-handedness. The exclusion criteria for all subjects comprised pathological brain magnetic resonance imaging (MRI) findings; inborn or other acquired diseases; any foreign implants in the body; BMI>32 or <19.8; present or past psychiatric or neurological disorders, substance dependency or substance abuse (including heroin, nicotine, or alcohol addiction); foreign implants in the body; any history of swing shift, night shift, or other shift work within the preceding year; any history of sleep complaints, or other sleep disorders, including hypersomnia, parasomnia, sleep related breathing disorder, sleep related movement disorder, or circadian rhythm sleep disorder, confirmed by overnight polysomnography; any history of significant head trauma or loss of consciousness >30 minutes; current smoking of more than 10 cigarettes per day; and consumption of >2 caffeinated beverages or potent tea per day.

### Research Design and Process

All volunteers participated voluntarily and were informed of the purposes, methods, and potential risks of this study, and signed an informed consent form. Volunteers were asked to complete a number of questionnaires, including the PSQI, Insomnia Severity Index (ISI), Self-Rating Scale of Sleep (SRSS), Self Rating Anxiety Scale (SAS), Self-Rating Depression Scale (SDS), HAMA, HAMD, and Profile of Mood States (POMS). The POMS questionnaire contains seven indices, including five negative emotion indices (nervousness, anger, fatigue, depression, and confusion) and two positive emotion indices (energy and self-esteem). An experienced psychiatrist evaluated the life histories of PIs with the Diagnostic and Statistical Manual of Mental Disorders, version 4 (DSM-IV) for the presence of psychiatric disorders, as well as an unstructured clinical interview for medical and sleep disorder history.

All volunteers underwent an fMRI scan, and seven PIs (3 male, 4 female) were scanned twice by MRI to examine the test-retest reliability. The interval between the two scans was 1–7 days.

### MRI

MRI scans were performed on 3-Tesla MR scanners (Trio, Siemens, Erlangen, Germany). High-resolution T1-weighted anatomical images were acquired with a three-dimensional spoiled gradient-recalled sequence in a sagittal orientation: 176 images (repetition time = 1900 ms, echo time = 2.26 ms, thickness = 1.0 mm, gap = 0.5 mm, acquisition matrix = 256 × 256, field of view = 250 mm × 250 mm, flip angle = 9°) were obtained. Finally, an 8-min rs-fMRI scan was obtained. A total of 240 functional images (repetition time = 2000 ms, echo time = 30 ms, thickness = 4.0 mm, gap = 1.2 mm, acquisition matrix = 64 × 64, flip angle = 90°, field of view = 220 mm ×  220 mm, 29 axial slices with Gradient-Recalled Echo-Planar Imaging pulse sequence) covering the whole brain were obtained.

A simple questionnaire was administered immediately after the approximately 3-min MRI scan to ask whether subjects were awake during the scan. Data from subjects who were asleep during scans were excluded.

### Data Analysis

MRIcro software (www.MRIcro.com) was used to ensure data quality. The first 10 time points of the functional images were discarded, due to the possible instability of the initial MRI signal and to allow the participants to adapt to the scanning environment. On the basis of MATLAB2010a (Mathworks, Natick, MA, USA), remaining data pre-processing was performed by Data Processing & Analysis for Brain Imaging (DPABI 2.1, http://rfmri.org/DPABI) toolbox, including Digital Imaging and Communications in Medicine standards for form transformation, slice timing, head motion correction and spatial normalization. Participants with more than 1.5 mm maximum translation in x, y, or z directions and 1.5° degree of motion rotation were rejected. The Friston 24 head motion parameters model was used to regress out head motion effects, based on recent work showing that higher-order models benefit from the removal of head motion effects^38,39^. Linear regression was applied to remove other sources of spurious covariates, along with their temporal derivatives, including the global mean signal, and the white matter and cerebrospinal fluid signal. After head-motion correction, functional MRI images were spatially normalized to the Montreal Neurological Institute (MNI) space and re-sampled at a resolution of 3 × 3 × 3 mm^3^. After preprocessing, the time series for each voxel were temporally bandpass filtered (0.01–0.1 Hz) and linearly detrended to reduce low-frequency drift and physiological high-frequency respiratory and cardiac noise and time series linear detrending.

### Calculation of Degree Centrality Maps

Degree centrality attributes a greater value to a voxel if it has strong connections with many other voxels in the brain. For the calculation of voxel-wise degree centrality, preprocessed fMRI data were used to perform voxel-based whole-brain functional correlation analysis. The Pearson’s correlation coefficients (r) between each pair of brain gray matter voxels were computed. As a result, we acquired a matrix of Pearson correlation coefficients depicting the whole-brain functional connectivity pattern. To obtain a graph for each subject, whole-brain functional network was then constructed by defining the threshold for each correlation^30,31^. Degree centrality was calculated by counting the number of significant suprathreshold correlations (or the degree of the binarized adjacency matrix) for each subject based on the individual voxel-wise functional network. Next, the voxel-wise degree centrality map for each individual was converted into a z-score map using the following equation:

$$Zi=\frac{{\rm{D}}{\rm{e}}{\rm{g}}{\rm{r}}{\rm{e}}{\rm{e}}\,{\rm{c}}{\rm{e}}{\rm{n}}{\rm{t}}{\rm{r}}{\rm{a}}{\rm{l}}{\rm{i}}{\rm{t}}{\rm{y}}\,-{\rm{m}}{\rm{e}}{\rm{a}}{\rm{n}}({\rm{D}}{\rm{e}}{\rm{g}}{\rm{r}}{\rm{e}}{\rm{e}}\,{\rm{c}}{\rm{e}}{\rm{n}}{\rm{t}}{\rm{r}}{\rm{a}}{\rm{l}}{\rm{i}}{\rm{t}}{\rm{y}}\,{\rm{o}}{\rm{f}}\,{\rm{a}}{\rm{l}}{\rm{l}}\,{\rm{v}}{\rm{o}}{\rm{x}}{\rm{e}}{\rm{l}}\,{\rm{i}}{\rm{n}}\,{\rm{b}}{\rm{r}}{\rm{a}}{\rm{i}}{\rm{n}}\,{\rm{m}}{\rm{a}}{\rm{s}}{\rm{k}}}{{\rm{s}}{\rm{t}}{\rm{d}}({\rm{D}}{\rm{e}}{\rm{g}}{\rm{r}}{\rm{e}}{\rm{e}}\,{\rm{c}}{\rm{e}}{\rm{n}}{\rm{t}}{\rm{r}}{\rm{a}}{\rm{l}}{\rm{i}}{\rm{t}}{\rm{y}}\,{\rm{o}}{\rm{f}}\,{\rm{a}}{\rm{l}}{\rm{l}}\,{\rm{v}}{\rm{o}}{\rm{x}}{\rm{e}}{\rm{l}}{\rm{s}}\,{\rm{i}}{\rm{n}}\,{\rm{b}}{\rm{r}}{\rm{a}}{\rm{i}}{\rm{n}}\,{\rm{m}}{\rm{a}}{\rm{s}}{\rm{k}}},$$

where *i* is the voxel index, degree centrality *i* is the degree centrality value for the *i* -th voxel, std is the standard deviation, and Z_*i*_ is the z-score for the *i*-th voxel. Finally, the resulting data was smoothed with a Gaussian kernel of 6 × 6 × 6 mm^3^ full-width at half-maximum.

In this study, we repeated the network analysis using a range of correlation r thresholds (i.e., r = 0.10, 0.15, 0.20, 0.25, 0.30, 0.35, 0.40, and 0.5) to determine whether between-group differences in degree centrality were substantially affected by the selection of different r-value thresholds or nodes used to construct brain networks.

### Seed-Based Connectivity Analyses

Brain regions with abnormal degree centrality were saved as seed points, and the average time series of these seed points were extracted from the residual image. Seed-based connectivity analyses were conducted to investigate the functional connectivity of these seed points with other voxels in the whole brain.

To make the data fit the normal distribution, we calculated the coefficient of Pearson correlation between ROIs and other voxels for the whole brain, and the resulting coefficient was subjected to Fisher’s Z transformation. To reduce the global effects of variability across the participants, the functional connectivity of each voxel was divided by the global mean value for each participant.

### Statistical Analyses

#### Behavioral Data

Comparisons of demographic factors (age, sex, and years of education) and sleep questionnaire data between PIs and GSs were performed using two-sample t-tests. Chi-square (χ^2^) test was used for categorical data. Statistical analysis was performed using IBM Statistical Package for the Social Sciences version 21.0 (SPSS 21.0). Data are presented as mean ± standard deviation. All the quoted results are two-tailed values, and P < 0.05 was considered statistically significant.

#### Voxel-Wise Degree Centrality

Before comparing the between-group differences in degree centrality, we first determined within-group statistical maps of degree centrality measurements for the PIs and GSs groups, calculated using one sample t-tests (P < 0.001, false discovery rate (FDR) corrected with a minimum continuous cluster voxel volume of 810 mm^3^). Then, two-sample *t*-tests were used to evaluate the voxel-wise differences in degree centrality in brain regions between PIs and GSs with age, sex, and years of education as nuisance covariates of no interest.

We analyzed between-group differences in degree centrality in two ways. First, we used thresholds of two-tailed voxel-wise *p* < 0.01 and cluster-level *p* < 0.01, corrected for multiple comparisons by FDR or Gaussian random field (GRF) theory. Second, once no between-group differences were found using the corrected thresholds, then we used a less stringent uncorrected statistical threshold of *p* < 0.01 with a minimum continuous cluster voxel volume of 810 mm^3^.

#### Multiple Linear Regression Analysis

Multiple linear regression analysis was performed to evaluate the relationships between behavioral factors (dependent variable) and degree centrality values of brain regions that differed between PIs and GSs (independent variable), and between behavioral factors (dependent variable) and strength of functional connectivity pairs of brain regions that differed between PIs and GSs (independent variable). P < 0.05 was considered significant.

## Results

### Sample Characteristics

The demographic characteristics of PIs are presented in Table 1. There were no significant differences between PIs and GSs in sex (p = 0.146), mean age (p = 0.758), mean education (p = 0.412), or PSQI time in bed (p = 0.199); however, compared with GSs, PIs had shorter PSQI total sleep time, lower PSQI sleep efficiency, higher PSQI scores, higher SRSS scores, higher SAS scores, higher SDS scores, higher HAMA scores, higher HAMD scores, higher POMS scores, higher scores for the five negative indices in POMS, and lower scores for the two positive indices in POMS (p < 0.001).

**Table 1:** Characteristics of PIs and GSs.

	PIs	GSs	t value	*p* value
**Demographics**				
Mean age, year	46.48 ± 12.6	45.69 ± 12.53	0.309	0.758
Sex (Male, Female)	48 (16, 32)	48 (23, 25)	2.116^#^	0.146^#^
Education, year	7.31 ± 4.03	7.96 ± 3.64	−0.824	0.412
**Sleep Questionnaires**				
Duration of insomnia, year	4.96 ± 5.25	N/A	N/A	N/A
Pittsburgh Sleep Quality Index (PSQI)	14.92 ± 2.04	2.56 ± 0.87	38.568	<0.001
PSQI total sleep time, hour	3.73 ± 1.31	7.27 ± 0.73	−16.337	<0.001
PSQI time in bed, hour	8.56 ± 1.1	8.33 ± 0.57	1.296	0.199
PSQI sleep efficiency, %	44.1 ± 16.44	86.91 ± 6.0	−16.944	<0.001
Self Rating Scale Of Sleep (SRSS)	34.94 ± 4.54	15.9 ± 1.51	27.555	<0.001
Insomnia Severity Index (ISI)	17.83 ± 2.94	1.54 ± 1.4	34.655	<0.001
Self-rating Anxiety Scale (SAS)	40.49 ± 6.84	27.96 ± 2.69	11.818	<0.001
Self-Rating Depression Scale (SDS)	47.68 ± 8.09	31.39 ± 3.06	13.057	<0.001
Hamilton Anxiety Scale (HAMA)	7.83 ± 3.39	1.88 ± 0.79	11.876	<0.001
Hamilton Depression Scale (HAMD)	9.06 ± 3.7	2.17 ± 1.08	12.402	<0.001
Profile of Mood States (POMS)	116.06 ± 20.81	82.98 ± 5.49	10.651	<0.001
Five negative index of POMS	29.52 ± 15.99	8.63 ± 3.26	8.87	<0.001
Two positive index of POMS	13.63 ± 7.84	25.65 ± 3.64	−9.631	<0.001

Note: Data are mean ± standard deviation values; ^#^, chi-square value; Self-rating Anxiety Scale and Self-Rating Depression Scale showed the standard score. The five negative index comprised nervousness, wrath, fatigue, depression and confusion, and the two positive index comprised energy and self-esteem.

Abbreviations: PIs, Patients with primary insomnia; GSs, Good sleepers; N/A, Not applicable.

### High Test-Retest Reliability Between Two MRI Scans

**Figure 1:**
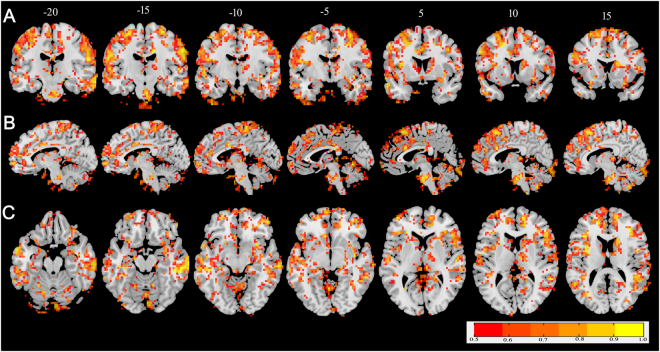
The intraclass correlation coefficients image thresholded at 0.5 or greater overlaid on the montreal neurological institute template brain in coronal (**A**), sagittal (**B**) and axial views (**C**). The color bar covers from red (ICC = 0.5) to yellow (ICC = 1).

The ICC result is illustrated overlaid on a structural template in in coronal (Fig. 1A), sagittal (Fig. 1B) and axial (Fig. 1C) views. The majority of brain voxels exhibited ICC values of 0.5 or greater. Fourteen clusters of at least 810 mm^3^ contiguous volumes with ICC values ≥ 0.8 were identified throughout the cortex, including in the right cerebellum posterior lobe, right pons, right superior temporal gyrus, right middle temporal gyrus, bilateral superior frontal gyrus, bilateral medial frontal gyrus, bilateral insula, right precentral gyrus, left postcentral gyrus and right precuneus.

### Binarized Differences in Degree Centrality

**Figure 2:**
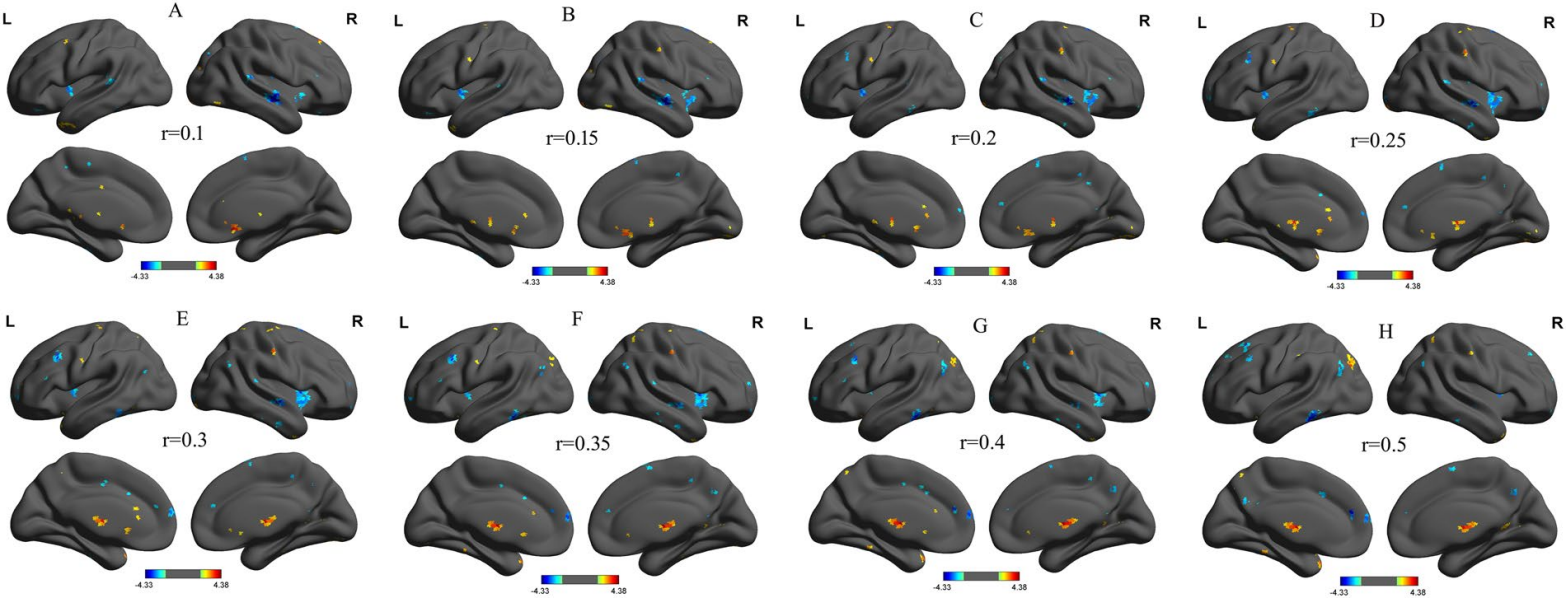
Binarized degree centrality mapswith several threshlod between PIs and GSs. Note: Betweent-group differences of binary network with different threshold at r = 0.10 (**A**), 0.15 (**B**), 0.20 (**C**), 0.25 (**D**), 0.30 (**E**), 0.35 (**F**), 0.40 (**G**) and 0.50 (**H**). Abbreviations:R, right; L, left; PIs, Patients with primary insomnia; GSs, Good sleepers.

We analyzed binary voxel-wise functional correlations and further investigated intra- and inter-group differences in binary voxel-wise functional brain centrality. We observed highly similar intra-group differences in binary degree centrality using several different thresholds (r = 0.10, Fig. 2A; 0.15, Fig. 2B; 0.20, Fig. 2C; 0.25, Fig. 2D; 0.30, Fig. 2E; 0.35, Fig. 2F; 0.40, Fig. 2G; and 0.50, Fig. 2H), indicating that the intra-group differences did not depend on the threshold used; therefore, we report only the results of binary network analysis using a threshold of r = 0.25.

**Figure 3:**
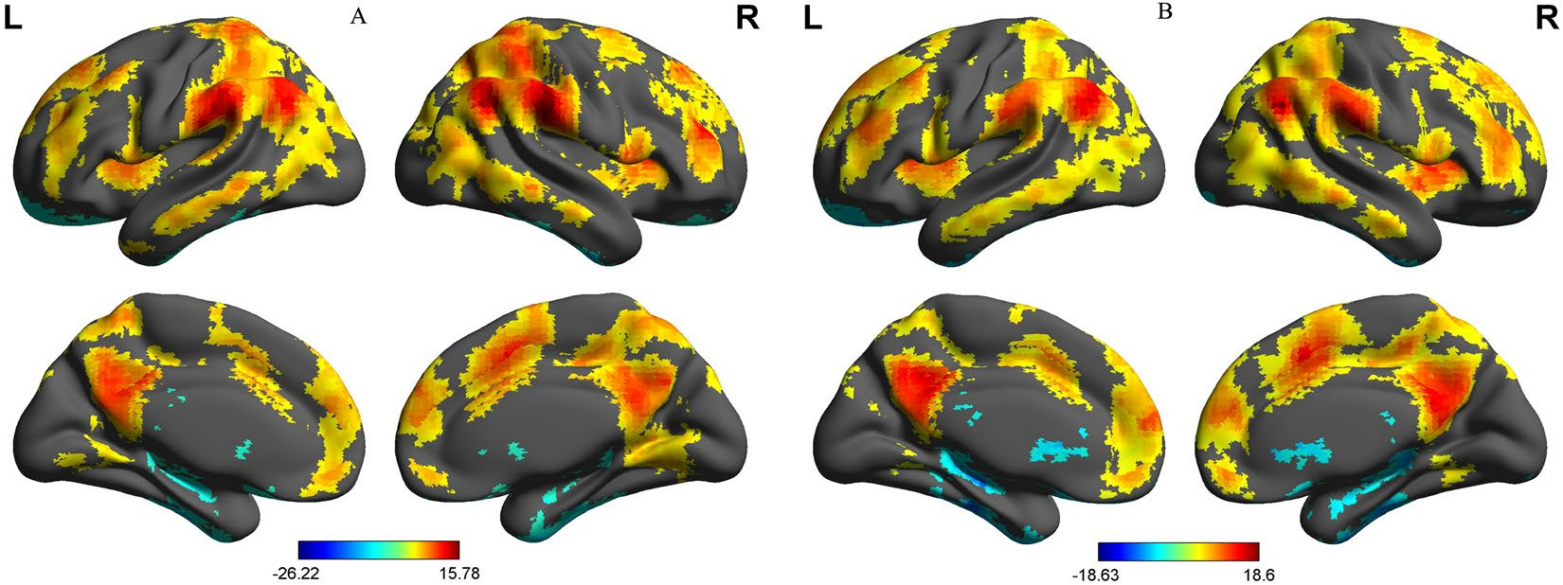
One sample t-test differences of PIs subjects (**A**) and GSs (**B**) in binarized degree centrality network with threshold at r = 0.25. Note: The statistical threshold was set at FDR corrected voxel threshold of p < 0.001 with a minimum voxel volume threshold of 810 mm^3^. Abbreviations: PIs, Patients with primary insomnia; GSs, Good sleepers; R, right; L, left.

Before comparing between-group degree centrality differences, we first constructed within-group statistical maps of degree centrality measurement for PIs (Fig. 3A) and GSs (Fig. 3B) groups separately, using one sample t-tests (P < 0.001, FDR corrected). We found that the two groups exhibited significantly similar differences in binarized degree centrality values in several brain networks, including the cerebellum, default mode network, visual network, and executive control network (Fig. 3A,B).

**Figure 4:**
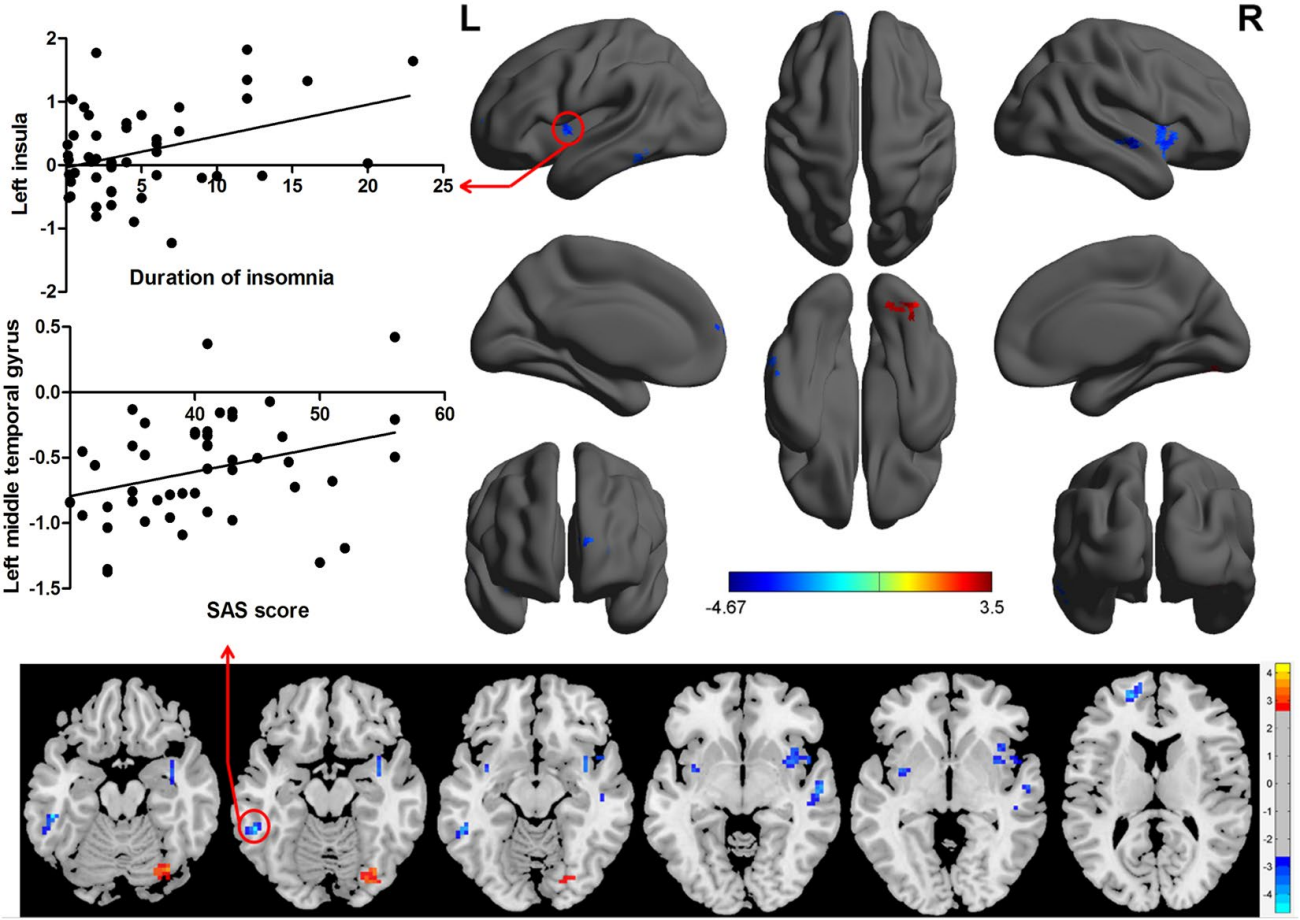
Between-group differences in binarized degree centralitynetwork with threshold at r = 0.25, and their correlations with behavioral performances in PIs. Abbreviations:R, right; L, left; SAS, Self-rating anxiety scale; PIs, Patients with primary insomnia.

We then conducted analysis of the binarized degree centrality patterns between PIs and GSs, thereby identifying inter-group differences in voxel-wise functional brain centrality. This analysis didn’t reveal any significant between-group differences after GRF or FDR correction. Using a more liberal uncorrected statistical threshold, the results of two-sample t-tests indicated significant inter-group differences in binary degree centrality networks in several related brain regions (P < 0.01, t = 2.63; Table 2, Fig. 4). Compared with GSs, PIs exhibited significantly higher degree centrality values in the right visual association cortex (BA19), extending to the right cerebellum posterior lobe, and significantly lower degree centrality values in the left middle temporal gyrus (BA20) in the executive control network, the right middle temporal gyrus (BA 22) in the auditory-language comprehension center, the bilateral insula (BA13) in the salience network, and the left medial prefrontal cortex (BA10) in the default mode network. As shown in Table 3 and Fig. 4, the duration of insomnia in PIs exhibited a positive linear correlation with degree centrality value in the left insula (R^2^ = 0.139, p = 0.009), while SAS score displayed a positive linear correlation with degree centrality value in the left middle temporal gyrus (R^2^ = 0.104, p = 0.026).

**Table 2:** The binarized degree centrality differences between PIs and GSs.

Conditions	Brain regions of peak coordinates	R/L	BA	Voxel volume (mm^3^)	t-score of peak voxel	MNI coordinates
						X, Y, Z
PIs>GSs	Occipital Lobe, Cerebellum Posterior Lobe	R	19	1350	3.4987	33 −72 −18
PIs<GSs	Middle Temporal Gyrus	L	20	945	−4.6669	−54 −42 −15
PIs<GSs	Middle Temporal Gyrus	R	22	864	−3.9199	54 −15 −6
PIs<GSs	Insula	L	13	999	−3.5931	−36 0 −3
PIs<GSs	Insula	R	13	2079	−3.6379	36 3 −12
PIs<GSs	Superior Frontal Gyrus	L	10	1188	−4.1077	−18 54 12

Notes: Between-group differences in binarized degree centrality thresholded at r=0.25. The statistical threshold was set at uncorrected voxel threshold of p<0.01 with a minimum voxel volume threshold of 810 mm^3^.

Abbreviations: PIs, patients with primary insomnia; GSs, good sleepers; R, right; L, left; BA, Brodmann’s area; MNI, montreal neurological institute.

**Table 3:** Multiple linear regression analysis between binarized degree centrality and behavioral performances in PIs.

Dependent variable	Independent variables	Coefficient (R^2^)	β	Standard error	t value	p value
Duration of insomnia	Left insula	0.139	2.804	1.028	2.727	0.009
SAS score	Left middle temporal gyrus	0.104	5.534	2.401	2.304	0.026

Abbreviations: PIs, patients with primary insomnia; SAS, Self-rating anxiety scale.

### Seed-Based Functional Connectivity Analysis

**Figure 5:**
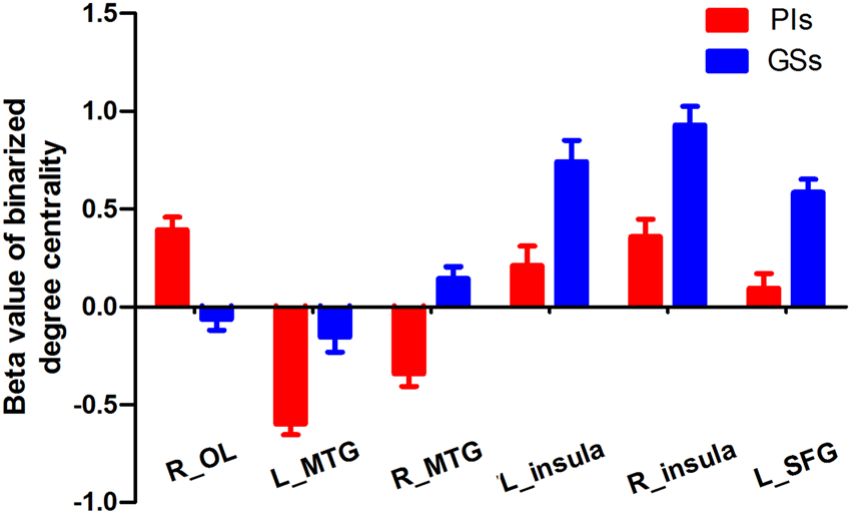
Binarized degree centrality value of between-group differences in different brain areas. Note: Data are mean ± standard error values. Abbreviations: PIs, Patients with primary insomnia; GSs, Good sleepers; R, right; L, left; OL, Occipital lobe; MTG, Middle temporal gyrus; SFG, Superior frontal gyrus.

**Figure 6:**
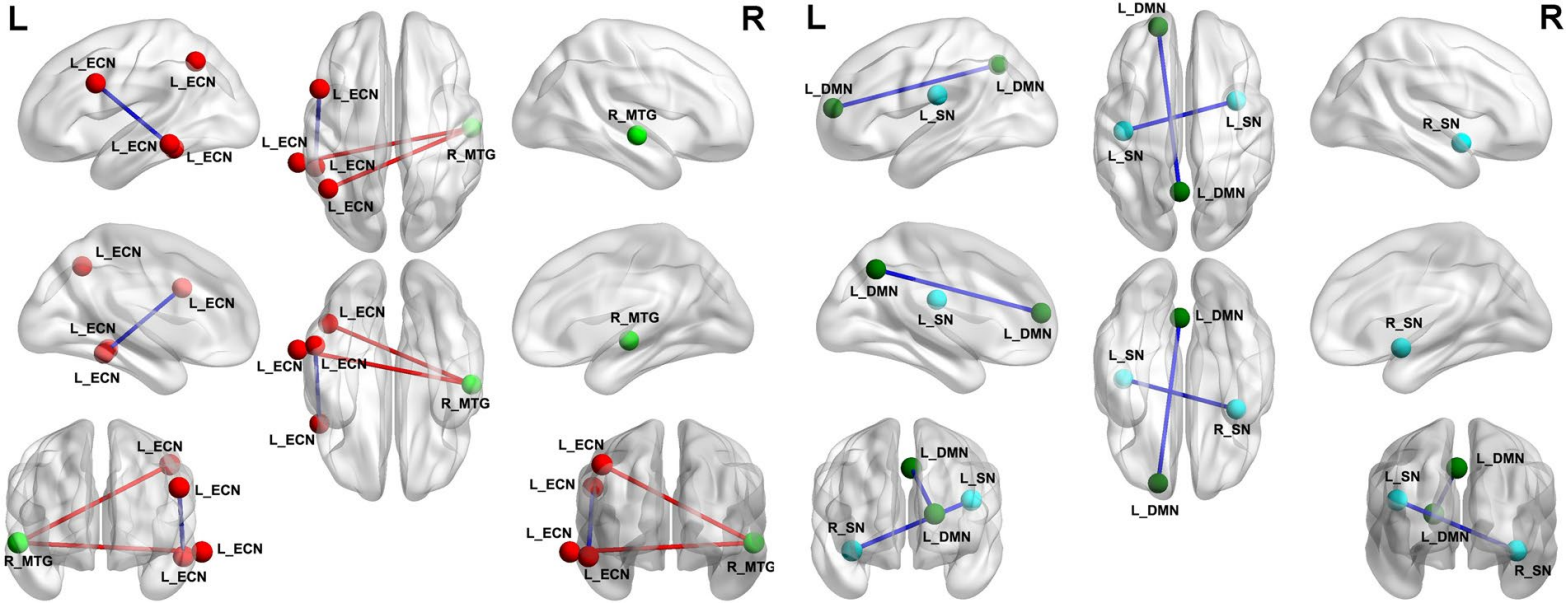
A holistic view of seed-based functional connectivity differences in intra- and inter-network between PIs and GSs. Note: Seed-based functional connectivity differences were found within the ECN, DMN and SN, and between the MTG and the ECN. Abbreviations: R, right; L, left; ECN, Executive control network; SN, Salience network; DMN, Default mode network; MTG, Middle temporal gyrus; PIs, Patients with primary insomnia; GSs, Good sleepers.

**Figure 7:**
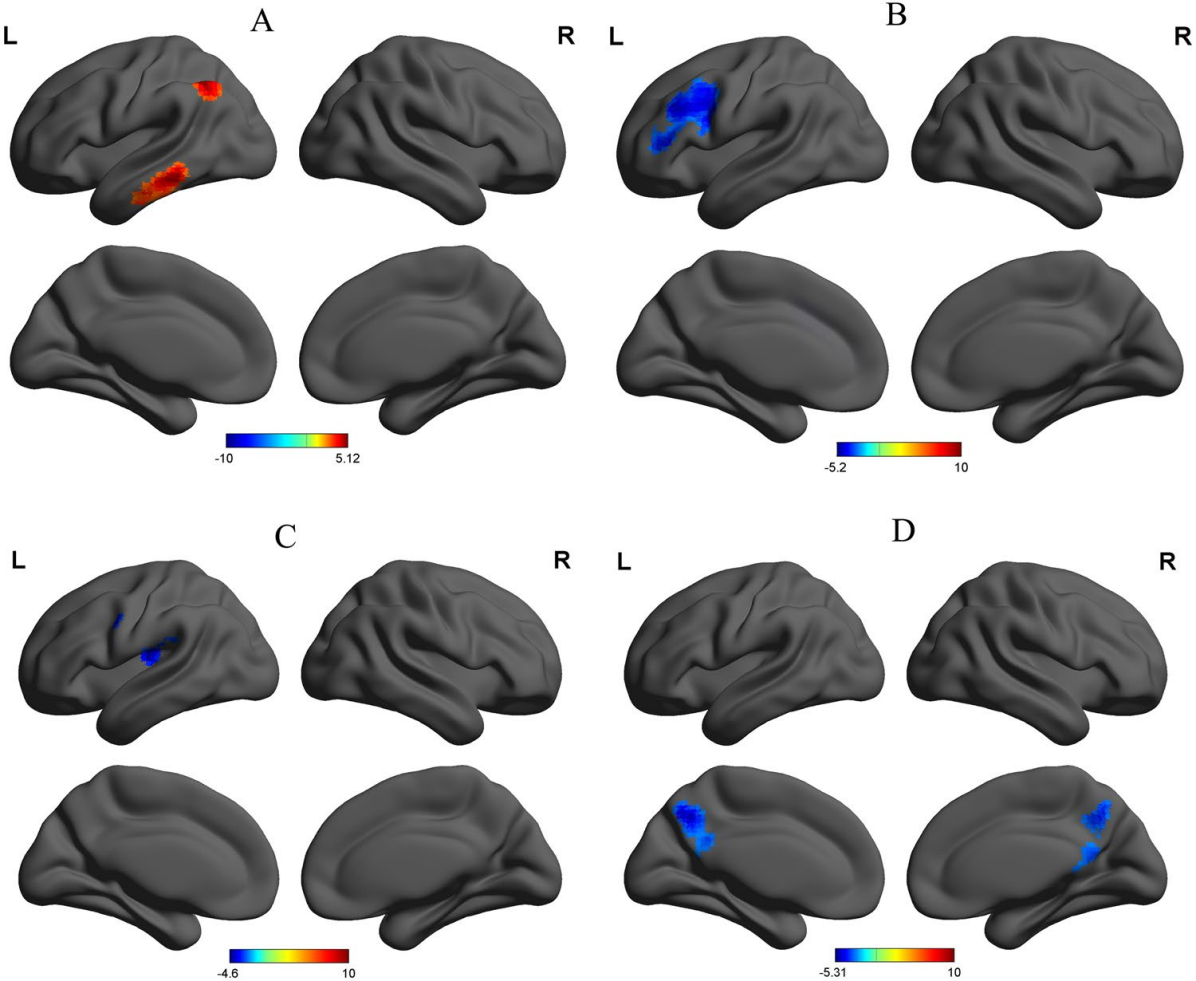
Seed-based functional connectivity differences for each region of interest between PIs and GSs. Note: Betweent-group differences in functional connectivity using the four ROIs that differ from binarized degree centrality, including right middle temporal gyrus (**A**), left middle temporal gyrus (**B**), right insula (**C**), and left superior frontal gyrus (**D**) with whole brain. Abbreviations: R, right; L, left; PIs, Patients with primary insomnia; GSs, Good sleepers; ROI, Region of interest.

The mean degree centrality values in different brain regions were extracted (Fig. 5). Then, those brain regions exhibiting abnormal degree centrality in PIs were selected as ROIs for further resting-state functional connectivity analyses. Two-sample t-tests revealed differences in resting-state functional connectivity between PIs and GSs (Table 4, Figs 6–7). When the left insula and the right cluster of the visual association cortex, extend to cerebellum posterior lobe, were used as seed-points, no significant between-group differences in functional connectivity were observed (p < 0.01, corrected by GRF). Using other seed points, several intra- and inter-network differences in seed-based functional connectivity were identified between PIs and GSs, including intra-network differences within the executive control network, default mode network, and salience network, and inter-network differences between the auditory language comprehension center and the executive control network (Table 4, Figs 6–7).

**Table 4:** Seed-based functional connectivity differences between PIs and GSs.

Seed point	Brain regions of peak coordinates	R/L	BA	Voxel volume (mm^3^)	t-score of peak voxel	MNI coordinates
						X, Y, Z
R_middle temporal gyrus	Middle temporal gyrus, inferior temporal gyrus	L	20,21	7155	4.9792	−66 −39 −12
	Inferior parietal lobule	L	7, 39, 40	7803	5.1241	−45 −57 45
L_middle temporal gyrus	Middle frontal gyrus, inferior frontal gyrus	L	9, 44, 45	18657	−5.204	−51 12 30
R_insula	Insula, Postcentral Gyrus	L	3, 13	8181	−4.6019	−42 −18 21
L_superior frontal gyrus	Precuneus	L	7	11259	−5.3114	−3 −60 42

Notes: Statistical threshold was set at voxel threshold of p < 0.01 and cluster threshold of p < 0.01with GRF correction.

Abbreviations: PIs, patients with primary insomnia; GSs, good sleepers; R, right; L, left; BA, Brodmann’s area; MNI, montreal neurological institute; GRF, Gaussian random field.

Compared with GSs, PIs exhibited a significant increace in functional connectivity between the right middle temporal gyrus in the auditory-language comprehension center and two clusters of the executive control network, including the left temporal lobe and left inferior parietal lobule (Table 4, Fig. 7A). The left middle temporal gyrus in the executive control network showed decreased functional connectivity with the left cluster of the middle frontal gyrus and inferior frontal gyrus in the executive control network (Table 4, Fig. 7B). The right insula in the salience network showed decreased functional connectivity with the left insula in the salience network (Table 4, Fig. 7C). The left superior frontal gyrus in the default mode network demonstrated decreased functional connectivity with the left precuneus in the default mode network (Table 4, Fig. 7D).

**Figure 8:**
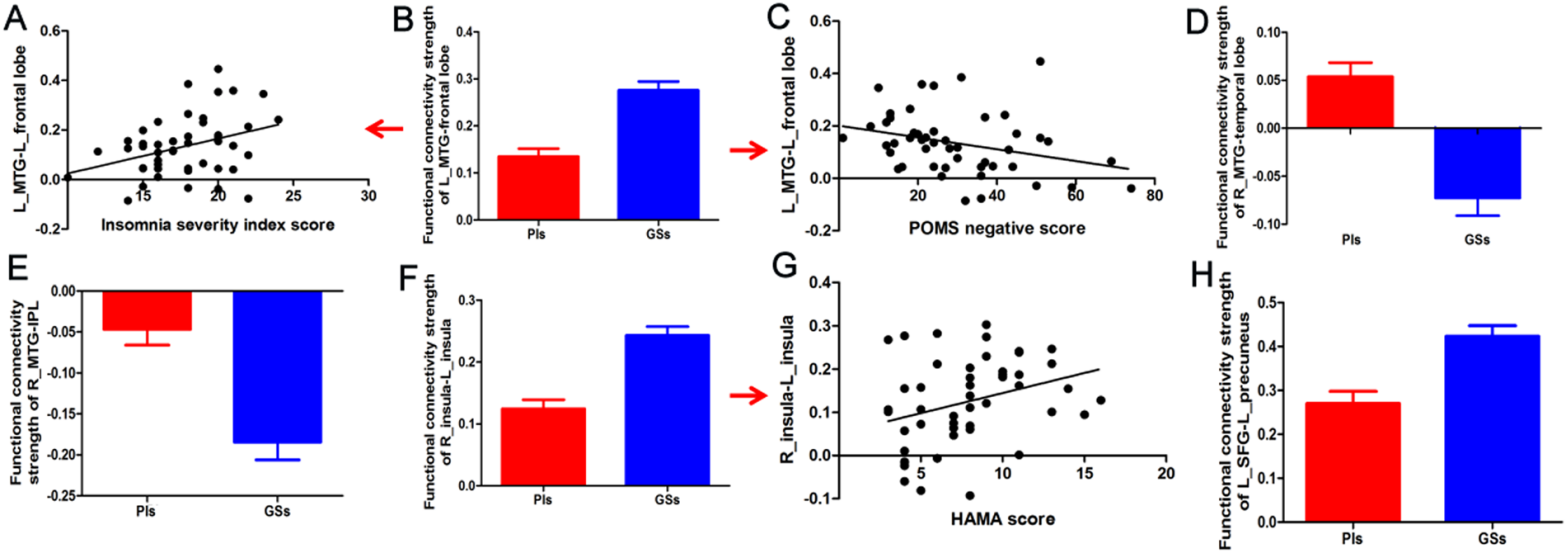
Binarized functional connectivity strength of between-group differences and their correlations with behavioral performances in PIs. Note: Data are mean ± standard error values. Abbreviations: R, right; L, left; MTG, Middle temporal gyrus; POMS, Profile of mood states; PIs, Patients with primary insomnia; GSs, Good sleepers; IPL, Inferior parietal lobule; HAMA, Hamilton anxiety scale; SFG, Superior frontal gyrus.

The strength of functional connectivity pairs of brain regions were then extracted and their linear correlations with behavioral factors analysed (Table 5, Fig. 8A–H). In PIs, the ISI score (Fig. 8A) and the five negative indices of POMS score (Fig. 8C) showed positive and negative linear correlation with the of functional connectivity pairs between the left middle temporal gyrus and the left frontal lobe (Fig. 8B) (R^2^ = 0.121, p = 0.015; R^2^ = 0.091, p = 0.037), respectively. The strength of functional connectivity pairs between the right and left insula (Fig. 8F) showed a positive linear correlation with HAMA score (Fig. 8G) (R^2^ = 0.097, p = 0.032).

**Table 5:** Multiple linear regression analysis between functional connectivity and behavioral performances in PIs.

Dependent variable	Independent variables	Coefficient (R^2^)	β	Standard error	t value	p value
Insomnia severity index	L_middle temporal gyrus-L_frontal lobe	0.121	8.596	3.411	2.52	0.015
Five negative index of POMS	L_middle temporal gyrus-L_frontal lobe	0.091	−40.521	18.864	−2.148	0.037
Hamilton anxiety scale	R_insula-L_insula	0.097	10.379	4.68	2.218	0.032

Abbreviations: PIs, patients with primary insomnia; POMS, Profile of mood states.

## Discussion

Degree centrality analysis reveals whole-brain network connectivity between a specific brain voxel and other brain voxels, rather than within specific nodes or networks, which is relatively high in the functional hubs of the brain network. Such analysis may serve as an important hub for information integration, superior information propagation, and critical way stations for information processing, leading to effective information flow^31,40^. To the best of our knowledge, the current study is the first to apply a resting-state degree centrality analysis approach to investigate the abnormal intrinsic functional hubs in PIs, and their relationships with behavioral factors. Furthermore, in our study, the degree centrality differences in regional brain areas showed a high test-retest stability between two MRI scans. PIs commonly complain of difficulties in initiating sleep at bedtime, frequent or prolonged awakenings, or early-morning awakening with an inability to return to sleep, which could be caused by the disruption of one or multiple functional brain networks. Thus, inefficient processing may arise from, or reveal disorganization of, one or multiple functional brain networks, leading to inefficiency in information transmission from one place to other places. Within this framework, in our study, PIs were associated with various behavioral factors and a sequential pattern of numerous changes in resting-state degree centrality indices of multiple intrinsic functional hubs, including higher degree centrality values in the right visual association cortex, and lower degree centrality values in the left executive control network, right auditory-language comprehension center, bilateral salience network, and left default mode network. Using these differences as ROIs, several intra- and inter-network seed-based functional connectivity differences were found between PIs and GSs, including intra-network differences in the executive control, default mode, and salience networks, and inter-network discrepancies between the auditory-language comprehension center and the executive control network. Furthermore, these intrinsic functional hubs exhibited linear correlations with behavioural features. Clarifying the biological mechanisms underpinning these functional connectivity outcomes could significantly advance our understanding of the neurobiological effects underlying insomnia.

Previous studies have suggested that the insula is a critical regions for sleep maintenance^41^. Recently, other studies identified regional alterations in brain activity^42,43^, and decreased functional connectivity^26,44^, brain morphometry^45^ and regional cerebral metabolism^46^ in the insula between PIs and GSs. Specially, the insula has been preferentially targeted, leading to an improved metabolic index after behavioral therapy^46^. These results highlight a potential role for the insula in insomnia. Reduced degree centrality values of brain functional hubs indicate lower levels of correlated activity and impaired roles of these hubs in facilitating neural network communication^47^. In support of these findings, in the present study, PIs exhibited significantly decreased degree centrality values in the bilateral insula, and decreased functional connectivity between the right insula and the left insula. These findings are consistent, and suggest that insomnia commonly causes disruption to the function and structure of the insula cortex. Insomnia is often comorbid with emotional disorders, and elevated emotional reactivity is thought to be an important factor in the etiology of insomnia^25^. In the present study, the duration of insomnia exhibited a positive linear correlation with degree centrality value in the left insula, while HAMA showed a positive linear correlation with the strength of functional connectivity between the right and left insula. These correlations were consistent with reports from a previous insomnia study, that insomniacs had involvement of greater blood oxygen level dependent level in the anterior insula with the salience networks, which was correlated with self-reported alertness and negative affect^43^. As a key hub of the salience network, the insular cortex integrates emotional and bodily states, and dysfunctional connectivity between this region and other brain areas may underlie changes in the cognitive function, vigilance, perception, subjective distress, poor sleep continuity, and interpersonal experience^43,48–52^. Abnormal activity of the insula is considered an important physical marker of pathological anxiety^53^; therefore, altered structure and function of the insula cortex may be associated with the underlying core neural mechanisms and dysfunctional emotional circuits in PIs.

The finding of decreased degree centrality values in the left superior frontal gyrus in the default mode network is not surprising. The prefrontal cortex has a major role in sleep physiology, including deactivation in rapid eye movement sleep^54^, and during the transition between wake and non-rapid eye movement^55^. Previous neuroimaging studies demonstrated reduced relative metabolism and altered cerebral response in the prefrontal cortex, and diminished performance relative to the prefrontal cortex function in PIs^56–58^. In this study, we found decreased functional connectivity between the left superior frontal gyrus and the left precuneus within the default mode network. The default mode network is associated with social cognitive processes related to decision making and self-regulation^59,60^. PIs showed lower deactivation of the default mode network regions in the prefrontal cortex with increased task difficulty, suggesting a functional disconnection in the prefrontal cortex may play a crucial role in the cognition dysfunction of PIs^58^. Structural alteration^61^ and aberrant functional connectivity^8,28,62,63^ within the default mode network have been reported by previous studies of primary insomnia and sleep deprivation. Consequently, our observation of reduced degree centrality value and functional connectivity within the default mode network may underlie pathophysiology of PIs.

We also found decreased degree centrality values in the left executive control network and decreased functional connectivity between the left and right executive control network in PIs. The executive-control network is involved in working memory, judgment, decision-making, and goal-directed behavior^64,65^, and has being identified as crucial for social anxiety disorder and anxiety^66,67^. Specifically, in the present study the SAS score displayed a positive linear correlation with degree centrality value in the left executive control network, and there were positive and negative linear correlations between the ISI and the five negative indices of POMS, respectively, and the strength of functional connectivity between the left and right executive control network. These data indicate that the disturbed connectivity patterns within the executive control network are involved in the regulation of negative emotions of PIs, and may be a key factor in the etiology of insomnia.

Previous physiological, neuroimaging, and neurocognitive studies have demonstrated ruminative, hypervigilant and/or excessive hyperarousal, and increased global cerebral metabolic rate for glucose utilization in primary insomnia^24,56,68–70^. The excess arousal refers to exaggerated cortical, somatic, and cognitive activation, which leads to increased sensory information processing and inability to initiate or maintain sleep^71^. The posterior middle temporal gyrus is proposed to store and provide access to lexical-semantic representations^72–75^. In addition to decreased degree centrality values in multiple brain regions, in the present study we also found significantly increased degree centrality in the visual association cortex, and increased functional connectivity between the right auditory-language comprehension center and the executive control network. The occipital lobe is primarily involved in the processing of visual information, and hyperarousal activation in the bilateral occipital gyrus can be observed during sleep deprivation status^7,76^ and in PIs^24^. A previous study demonstrated that normal activation of the auditory cortex is decreased to help maintain sleep in response to external stimuli^77^; however, our observation of increased functional connectivity between the right auditory-language comprehension center and the executive control network may highlight a reduced capacity to disengage from information processing of external auditory stimuli, which is consistent with the clinical characteristics of PIs, who have shallow sleep and increased sensitivity to the surrounding environment. Our data therefore support the theory of hyperarousal, and provided evidence that the hyperarousal model may be a core predisposing or perpetuating factor in primary insomnia^24^.

## Conclusions

Our study provides new insights into the dysfunction and pathophysiology of insomnia using a graph-theoretic measurement, unbiased opportunity to search for abnormalities within the entire connectivity matrix of the full-brain functional connectome without a priori hypothesis. The results reveal a pattern of functional deficits in multiple core networks, which appear to be disrupted in PIs, including at least three principal neural systems: the executive control, salience, and default mode networks. These disturbed networks were correlated with the negative emotions and insomnia severity in the PIs group. Altered connection properties of important network hubs in the executive control, salience and default mode networks may be neural risk factors for neuropsychological changes underlying emotional and cognitive impairments in primary insomnia. The findings of this study highlight the role of functional connectivity in the pathophysiology of PIs and broaden our understanding of the functional characteristics of PIs. Clarification of the biological mechanisms underlying these alterations in functional connectivity could significantly advance our understanding of the neuropsychological changes involved in insomnia and may facilitate determination of the neural mechanisms underlying behavioral impairments in individuals with insomnia.

One of the strengths of the present study is the relatively large sample size; however, there are several limitations that should be noted. First, there were no measure of cognitive function in the present study. It’s difficult to draw a defined conclusion about the relationship between the default mode network and the cognitive function. Second, our findings are limited by the use of the Fitbit Flex tracker to monitor the sleep quality in our experience^24^. Although we cannot provide direct evidence to prove whether the FITBIT tracker provides a valid and reliable measure of objective sleep, we compared some patients’ data between the FITBIT and the PSG, and found the results were similar. In fact, our sample was screened to exclude individuals with medical or psychiatric disorders that may affect sleep, and the diagnosis of PIs mainly depends on the experience of senior physicians who have been working for more than 20 years. Third, the PIs had clinically significant scores on the HAMD scale which can potentially confound the results. Although anxiety, depression and insomnia are comorbidities, future studies should recruit a larger sample size to explore the functional connectome differences in PIs with or without anxiety and depression symptom.
